# Effects of silybin on triptorelin-induced bone metabolic abnormalities in prostate cancer revealed based on TMT-based proteomics

**DOI:** 10.1371/journal.pone.0341064

**Published:** 2026-01-29

**Authors:** Jianhui Li, Xuejiao Lv, Ying Jiang

**Affiliations:** 1 Heilongjiang University of Chinese Medicine, Harbin, China; 2 College of Basic Medicine, Heilongjiang University of Chinese Medicine, Harbin, China; Purdue University, UNITED STATES OF AMERICA

## Abstract

**Aims:**

The aim of this study was to investigate the mechanism of action of SB on TRP-induced abnormalities of bone metabolism in LNCaP cells.

**Methods:**

The effects of different concentrations of SB and TRP alone and in combination on the proliferation of LNCaP cells were examined by CCK-8 assay, and the half maximal inhibitory concentration were screened for the subsequent experiments. Transwell migration and invasion assays were used to further investigate the effects of SB and TRP alone and in combination on the migration and invasion ability of LNCaP cells. Based on Tandem Mass Tag (TMT) labeling and liquid chromatography-tandem mass spectrometry (LC-MS/MS) technology, the differentially expressed proteins (DEPs) of LNCaP cells in the control group, SB group, TRP group and combination group were quantified. Bioinformatics technology was used to analyze the DEPs of LNCaP cells in each group. At last, the achieved key targets were verified by western blot.

**Results:**

The inhibitory effects of different concentrations of SB and TRP alone and in combination on the proliferation, migration and invasion of LNCaP cells showed both time-dependent and concentration-dependent effects, and the inhibitory effects of the combination of drugs on LNCaP cells were more significant. The proteomics results showed that a total of 153 DEPs were identified in the SB group and the control group, 100 DEPs were identified in the TRP group and the control group, and 524 DEPs were identified in the combination group and the control group.

Bioinformatics analysis showed that a higher number of DEPs were enriched in the IL-17 signaling pathway in the SB and combined treatment groups compared to the control group. These findings suggest a potential role of SB and the combined treatment in modulating the IL-17 signaling pathway. In contrast, the same DEPs were found to be enriched in both the Circadian entrainment and Apelin signaling pathways in the TRP versus control and combined treatment versus control comparisons.

**Conclusions:**

SB may regulate TRP-induced bone metabolism abnormalities in LNCaP cells through the IL-17 signaling pathway as well as five DEPs: p-ERK2, RELA, HSP90B1, GNAI1and GNAI3.

## 1. Introduction

Prostate cancer (PCa) is the most common male genitourinary malignancy [1], which seriously threatens the survival of older men [2]. Worldwide, the incidence and mortality of PCa are increasing year by year [3–5]. Although the molecular mechanisms of prostate cancer occurrence, development and metastasis have not been fully revealed, age, race, geographical distribution, diet and family history are important factors contributing to the development of the disease [6–8]. At the present stage, the common treatment for prostate cancer includes prostatectomy, radiotherapy, chemotherapy, androgen receptor (AR) antagonists, novel endocrine therapy, and immunotherapy [9,10]. However, there are still a considerable number of patients with poor prognosis such as recurrence and metastasis after surgery [11], which will shorten their survival and reduce their quality of life [12]. PCa bone metastasis is a common complication in advanced PCa, typically involving disruption of the bone microenvironment and abnormal bone metabolism [13,14]. Upon entering the bone marrow cavity, circulating cancer cells first adapt to the skeletal microenvironment, remaining dormant for an extended period. At a specific time point, these cells transition from dormancy to an active proliferative state. Ultimately, PCa invasion disrupts the osteogenic-osteolytic balance, leading to abnormal bone formation and accelerating the progression of PCa [15,16]. Therefore, further research on the mechanisms of prostate cancer development and the development of more effective drugs and treatments with fewer side effects are still urgent issues.

Silybin (SB) is the main constituent extracted from the seeds of Asteraceae silybum marianum, which has high antioxidant and anticarcinogenic properties, as well as broad-spectrum anticancer efficacy [17]. SB also has other therapeutic effects such as hepatoprotective, antidiabetic, and cardiovascular protection [18]. The anticancer properties of SB have been demonstrated in a range of cancer models such as prostate, lung, colon, breast, bladder and hepatocellular carcinoma [19]. SB can also affect prostate cancer-related growth factors [20], cell cycle regulators and vascular endothelial growth factor [21], realizing an inhibitory effect on cell growth as well as mitosis, or inhibiting cell cycle progression [22], which is important for prostate cancer prevention as well as treatment. Some other findings suggest that SB has a positive effect on the regulation of bone metabolism, and SB can attenuate the downstream signaling cascade associated with RANKL and TNF-α to inhibit osteoclastogenesis [23], suggesting its potential for the treatment of osteoporosis and other bone diseases.

Triptorelin (TRP) is a synthetic gonadotropin-releasing hormone (GnRH) agonist with a molecular formula of C_64_H_82_N_18_O_13_ [24]. TRP is currently used in the treatment of prostate cancer [25], precocious puberty [26], endometriosis [27] and uterine fibroids [28]. In the process of treating prostate cancer, TRP acts on the hypothalamic-pituitary-gonadal axis, and through chronic stimulation it will down-regulate the pituitary GnRH receptor, inhibit the body’s production of luteinizing hormone and follicle-stimulating hormone, and lead to a decrease in the secretion of testosterone and estrogen [24], so as to achieve the purpose of treating locally advanced and metastatic prostate cancer, and TRP’s efficacy of its action in vitro and in vivo is higher than that of natural GnRH by a factor of 100-fold [29]. Therefore, in 2000, TRP was approved for the treatment of prostate cancer in the United States, and to date, it is still used as a first-line therapeutic agent for hormone-responsive malignancies [30]. TRP produces some side effects in the treatment of prostate cancer, including osteoporosis, hot flashes, erectile dysfunction, weight gain, and female-like breast development [31–33]. These side effects seriously affect the quality of life of patients.

In the clinical treatment of prostate cancer, combination therapies are increasingly recognized for their ability to enhance efficacy, reduce side effects, and improve overall patient outcomes. While various drug combinations, such as docetaxel and thymoquinone, metformin and atorvastatin, and GLI-ANTagonist 61 and metformin, have shown enhanced antitumor effects and higher sensitivity compared to individual drugs [34–37], the rationale for combining SB with TRP lies in the unique role of SB in modulating bone metabolism. TRP, while effective in prostate cancer treatment, is associated with significant adverse effects on bone metabolism, leading to bone loss and osteopenia [38–40]. SB has demonstrated the ability to modulate key pathways involved in osteoclastogenesis, bone homeostasis, and inflammation [23,41,42], making it a promising candidate to mitigate TRP-induced bone metabolic abnormalities. Previous studies have shown that SB can inhibit osteoclast differentiation and activity [43,44], and there is growing interest in the possibility that it may help protect against bone loss associated with cancer therapies. Therefore, the combination of SB with TRP may offer a novel approach to not only enhance the efficacy of prostate cancer treatment but also reduce the bone metabolic side effects associated with TRP.

In the present study, we used LNCaP cells cultured in vitro to explore the effect of SB combined with TRP on their bone metabolism, and used Tandem Mass Tag (TMT) labeling and liquid chromatography-tandem mass spectrometry (LC-MS/MS) technology to analyze the differentially expressed proteins (DEPs) of LNCaP cells cultured in the control group, the SB group, the TRP group, and the combination group under different conditions. We also performed bioinformatics analysis of the DEPs under the four conditions. The DEPs and signaling pathways identified in this study may be important in SB inhibition of TRP-induced bone metabolism abnormalities in LNCaP cells, further promoting the use of SB combined with TRP in the treatment of PCa.

## 2. Materials and methods

### 2.1 Drug

SB and TRP were purchased from Macklin, and they were diluted with androgen-free medium (RPMI-1640 medium + 10% androgen-free serum + 1% P/S). Different concentrations of the drugs were prepared separately, four concentration gradients each for SB (50, 100, 150, 200 μmol/L) and TRP (50, 100, 150, 200 μmol/L).

### 2.2 Cell culture

The human prostate cancer cell line (LNCaP) was purchased from Otwo Biotech, and the cells were cultured in normal medium (RPMI-1640 medium + 10% FBS + 1% P/S) at 37 °C in a 5% CO_2_ incubator, and the medium was changed every 48h. When the cells were in logarithmic growth phase and the density was about 90%, the cells were passaged. The third generation of cells will be used for subsequent experiments.

### 2.3 Cell viability analysis

Cell viability assay kit (CCK-8) was used to detect the proliferation ability of LNCaP cells. LNCaP cells with good growth status were taken, digested with trypsin, and normal medium was diluted into single-cell suspension, and the cell density was adjusted to 1.5 × 10^4^ cells per milliliter. The cell suspension of 200 μL per well was inoculated into 96-well plates with 6 replicate wells in each group, and the peripheral ring of edge wells was filled with equal amount of sterile PBS. Cells were attached to the wall 48 hours after inoculation, and the medium in the wells was discarded and replaced with androgen-free medium for another 48h. Then, the culture medium was changed into different drug-containing androgen-free medium, which were: null group (androgen-free medium without cells); control group (androgen-free medium with cells); SB group (50, 100, 150, 200 μmol/L); and TRP group (50, 100, 150, 200 μmol/L); SB and TRP combination group (SB 200 μmol/L and TRP 100 μmol/L; SB 100 μmol/L and TRP 200 μmol/L). After 48h of drug intervention, the old medium was replaced with CCK-8 solution and androgen-free medium in the ratio of 1:9, and the cells were placed in an incubator to continue incubation for 2h, and the absorbance (A) was measured at 450 nm with an enzyme meter, and the measurement was repeated three times for each group to calculate the proliferation rate. The formula for calculating proliferation rate was: proliferation rate = [(As-Ab)/ (Ac-Ab)] ×100%, As represents the test wells, Ac represents the control wells, and Ab represents the blank wells.

### 2.4 Wound healing assay

The cells in each group were cultured until the density was fused. The medium was changed to serum-free medium and treated with 1 μg/mL of mitomycin C for 1 h before the experiment. Then, 200 μL pipette tip was utilized to scratch perpendicular to the cell plane on the cell layer of each group, and then the cell surface was washed with serum-free medium for 1 time to remove cell debris. The cells in each group were placed in an incubator at 37°C and 5% CO2, and were photographed and recorded after incubation for 0 h, 24 h and 48 h using drug-containing medium, respectively.

### 2.5 Transwell migration and invasion assay

Transwell chambers were used to assess the migration and invasion ability of LNCaP cells. LNCaP cells with good growth status and in logarithmic growth phase were taken and resuspended in serum-free medium, and then 100 μL of cell suspension was added to the upper chamber of transwell chambers. And drug-containing medium was added to the upper chamber of the transwell chamber corresponding to each group: control group (100 μL of serum-free medium); SB group (100 μL of 100 μmol/L SB serum-free medium); TRP group (100 μl of 200 μmol/L TRP serum-free medium); and the combination of SB and TRP group(100 μl of 100 μmol/L SB serum-free medium and 100 μl of 200 μmol/L TRP serum-free medium). The migratory ability of LNCaP cells was stimulated by adding 600 μL of serum-containing medium to the lower chamber of the transwell. After 48 h of culture, cells that crossed the membrane were fixed with paraformaldehyde and stained with crystal violet for 30 min. Cells were observed under a light microscope and photographed for recording.

The diluted matrix gel was added to the bottom of the upper chamber of the transwewll chambers. LNCaP cells in good growth condition and in logarithmic growth phase were taken and resuspended in serum-free medium, and then 100 μL of the cell suspension was added to the upper chamber of the transwell. The rest of the operation was performed as above to evaluate the invasive ability of LNCaP cells.

### 2.6 Treatment and grouping of LNCaP

Based on the results of previous experiments, LNCaP cells were divided into four groups for subsequent proteomics analysis: group A: cells were cultured with androgen-free medium; group B: cells were cultured with androgen-free medium containing 100 μmol/L SB; and group C: cells were cultured with androgen-free medium containing 200 μmol/L TRP; group D: cells were cultured with androgen-free medium containing 100 μmol/L SB and 200 μmol/L TRP.

### 2.7 Proteomics

#### 2.7.1 Protein extraction and quantification.

Proteins were extracted from each group of samples and processed separately. To each sample, an appropriate amount of lysis buffer was added, dissolved and mixed by vortexing, centrifuged at 14,000g for 20 min, the supernatant was collected, 10uL was taken for quantification, and the rest was frozen at −80°C. Protein concentration was determined using BCA protein assay kit.

#### 2.7.2 TMT labeling and fractionation.

An appropriate amount of purified protein extract was taken from each sample and reduced with dithiothreitol of 5 mmol/L for 1h at 37°C and returned to room temperature. Subsequently, 10 mmol/L iodoacetamide was added for alkylation for 45 min at room temperature and protected from light, and finally the samples were diluted 4-fold with 25 mmol/L ammonium bicarbonate. Trypsin was added in the ratio of trypsin: protein = 1:50, and the digestion was carried out overnight at 37°C, and the enzyme digestion was terminated the next day by adding formic acid to adjust the pH to be less than 3. Samples were desalted with C18 Zip Tips, 100% acetonitrile activated desalting column, 0.1% formic acid equilibrated column, samples were loaded onto the column, the column was washed with 0.1% formic acid to wash out impurities, and finally eluted with 70% acetonitrile, and flow-through solution was collected and freeze-dried.

According to the manufacturer’s instructions, the samples were mixed with TMT-labeled reagent (TMT10-plex) and left to react at room temperature for 1 h. Finally, the reaction was terminated by adding hydroxylamine to the samples. The differentially labeled samples were homogeneously mixed, vortexed and shaken, centrifuged to the bottom of the tube, and then vacuum freeze-dried.

The TMT-labeled peptide mixture was dissolved with 100 μl of mobile phase A, followed by centrifugation at 14,000 g for 20 min, and the supernatant was extracted, and then the peptides were separated by high-pH reverse-phase HPLC using a chromatographic column (XBridge Peptide BEH 5um C18 Column) at a flow rate of 0.7 ml/min, with the temperature was 50°C. Mobile phases A (2% acetonitrile, pH adjusted to 10.0 using ammonium hydroxide) and B (98% acetonitrile, pH adjusted to 10.0 using ammonium hydroxide) were used to create a gradient elution. The gradients were set as follows: 5–8% B, 0–5 min; 8–18% B, 5–40 min; 18–32% B, 40–62 min; 32–95% B, 62–64 min; 95% B, 64–68 min; 95–5% B, 68–72 min. All fractions were freeze-dried and used for subsequent analysis.

#### 2.7.3 LC-MS/MS analysis.

Mobile phase A was an aqueous solution containing 0.1% formic acid and 100% water, and mobile phase B was an aqueous solution containing 0.1% formic acid and 80% acetonitrile. The peptide lyophilized powder was dissolved in 10 µL of mobile phase A solution and centrifuged at 14,000 g for 20 min at 4°C, then 1 µg of the supernatant sample was taken for separation. The liquid phase gradient was set as follows: 6%B,0–8 min; 6–15%B, 8–15 min; 15–25%B, 15–42 min; 25–40%B, 42–57 min; 40–100%B, 57–58 min; 100%B, 58–68 min; 100–6%B, 68–69 min; 6%B, 69–85 min. The flow rate was maintained at 300nL/min.

The peptides were separated by high performance liquid chromatography (HPLC) and exposed to a nanospray ionization (NSI) source, and then entered into an ORBITRAP ECLIPSE mass spectrometer for tandem mass spectrometry (MS/MS). The applied electrospray voltage was 2.0 kV, the compensation voltage was set to switch between −45 V and −65 V every 1 S, the ion transfer tube temperature was set to 320 °C, and the data acquisition mode used the data-dependent acquisition (DDA) program. The full scan range was set to 350–1500 m/z, the primary MS resolution was set to 120,000 (200 m/z), the automatic gain control (AGC) was set to 4E5, and the maximum injection time (MIT) was set to 50 ms. After the parent ions were fragmented using the higherenergy collision dissociation (HCD) method, a secondary scan was performed with the resolution set to 30,000 (200 m/z), AGC set to 5E4, MIT set to 54 ms, and the peptide fragmentation collision energy set to 36%. The ion dynamic exclusion time was set to 30 s to avoid repeated scanning of the parent ion. MS raw data were generated.

#### 2.7.4 Database search.

MS raw data were quantified and explored using Proteome Discoverer 2.4 (Thermo Fisher Scientific, Waltham, MA, USA) and UniProt Homo Sapiens database (http://www.uniprot.org). Various search parameters were set as follows: the enzyme digestion method was set to Trypsin; the maximum number of missed cleavages was set to 2; the minimum length of the peptide was set to 7 amino acid residues; and the mass tolerance was set to 15 ppm for primary parent ions and 0.02 Da for secondary fragment ions. Carbamidomethyl (C) was set as a fixed modification and oxidation of methionine, Acetyl (Protein N-terminal), TMT-10plex (K, N-terminal) was set as a variable modification. The quantification method was set to TMT-10plex. the False discovery rate (FDR) for protein, peptide, and spectra identification were all set to 1%. DEPs were screened by Student’s *t*-test *p*-value <0.05 and fold change (FC)>1.2 or FC < 0.83, and were defined as up-regulated when FC > 1.2 and down-regulated when FC < 0.83.

#### 2.7.5 Data quality analysis.

The quality of proteomics data was assessed by heat map analysis, principal component analysis (PCA) analysis, relative standard deviation analysis and normalized expression analysis.

### 2.8 Bioinformatics analysis

Functional enrichment analysis of differentially expressed proteins was performed using the Gene Ontology (GO) database, and the identified proteins were categorized into the following three groups: biological process (BP), cellular component (CC), and molecular function (MF). Signal-passage enrichment analysis of DEPs was performed using the Kyoto Encyclopedia of Genes and Genomes (KEGG) database (https://www.genome.jp/kegg/). Protein-protein interaction (PPI) networks of DEPs were obtained through the STRING database (http://www.string-db.org/).

### 2.9 Western blot analysis

Groups of samples were lysed with lysis solution, centrifuged and the supernatant was taken. Protein concentration was determined using BCA protein assay kit. In addition, 40 μg of each sample was separated by 10% SDS-PAGE and the proteins were transferred to PVDF membranes. Then, the membranes were blocked with 5% skimmed milk in TBST for 1 h. The membranes were then incubated with the following primary antibodies: p-ERK1/2 antibody (1:1000), HSP90B1 antibody (1:500), GNAI1 antibody (1:500), GNAI3 antibody (1:500) at 4°C overnight. After washing with TBST 4 times for 5 min each, the gel was incubated with secondary antibody: sheep anti-rabbit IgG-HRP (1:5000) for 45 min at 37°C. Finally, the reaction was left to react with ECL luminescent solution for 5 min. and covered with a plastic sealing film, and exposure was performed in a dark room. The optical density values of the target bands were analyzed with Gel-Pro-Analyzer software.

### 2.10 Statistical analysis

Statistical analysis was performed with Statistical Program for Social Sciences (SPSS) (SPSSInc., version 20.0, United States). All charts were generated using GraphPad Prism 8.0.2. Each experiment was repeated more than three times. The quantitative data were reported as the means ± Standard Deviation (SD), and the significant difference was analyzed with Student’s *t*-test between two groups, one-way analysis of variance (ANOVA) was used for comparisons among multiple groups, *p*-value <0.05 was considered statistically significant. In TMT proteins with *p*-value <0.05 and fold changes (FC) > 1.2 or < 0.83 were considered as DEPs. GO and KEGG analyses were carried out using Fisher’s exact test, using the entire quantified protein annotations as the background dataset. Categories and pathways with *p*-value <0.05 were considered statistically significant.

## 3. Results

### 3.1 Silybin, triptorelin alone and in combination inhibit the proliferation of LNCaP cells

**Fig 1:**
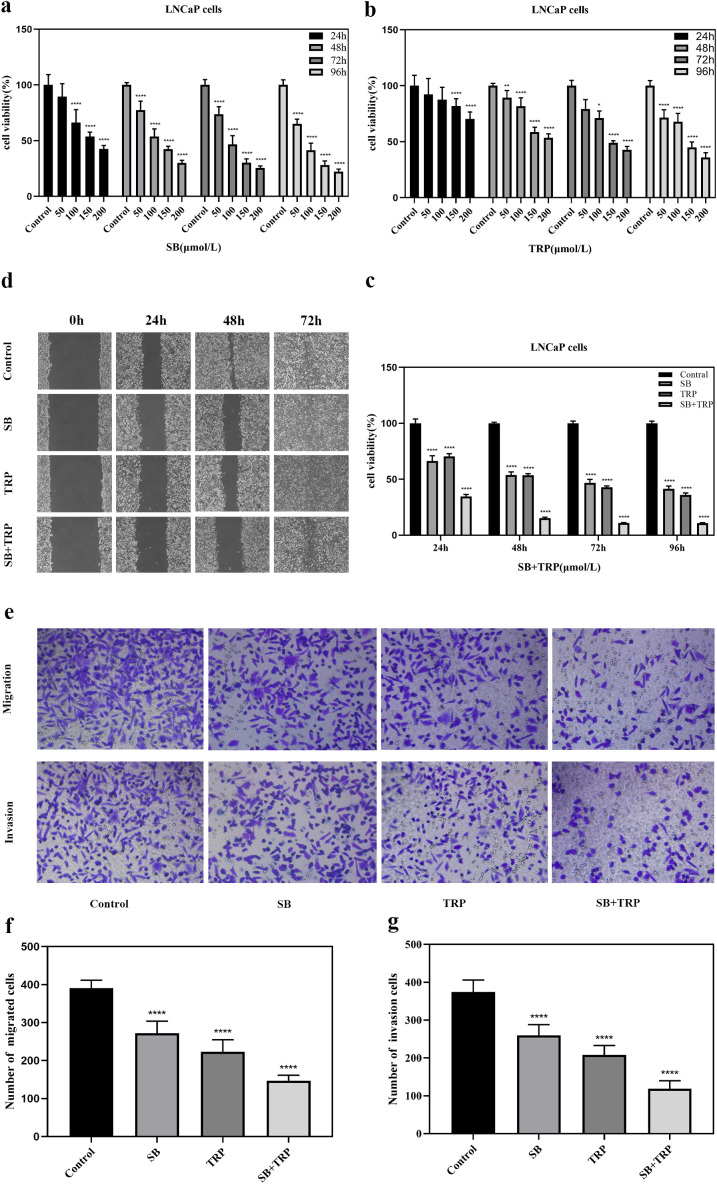
The effect of SB and TRP on the viability, migration, and invasion of LNCaP cells. **(a)** and **(b)** LNCaP cells were treated with different concentrations of SB (50, 100, 150, or 200 μmol/L) or TRP (50, 100, 150, or 200 μmol/L) for 24, 48, 72, and 96 h. Cell viability was determined by CCK-8 assays. **(c)** LNCaP cells were treated with 100 μmol/L SB and 200 μmol/L TRP for 24, 48, 72, and 96 h. Cell viability was determined by CCK-8 assays. **(d)** The effect of each group (Control, SB, TRP, and SB + TRP) on LNCaP cell migration was detected by a wound healing assay. **(e)**, **(f)**, and **(g)** The effect of each group (Control, SB, TRP, and SB + TRP) on LNCaP cell migration and invasion was detected using Transwell chambers. The data are shown as the mean ± SD values; *P < 0.05, **P < 0.01, ****P < 0.0001, vs. untreated control group.

To investigate the effects of SB and TRP alone and in combination on the proliferation of LNCaP cells, the viability of LNCaP cells was determined by CCK-8 assay. The results showed that the viability of LNCaP cells was dose-dependent and time-dependent by SB and TRP (Fig 1a-1b), and similarly dose-dependent and time-dependent by the combination of the drugs (Fig 1c). This indicated that SB and TRP alone and in combination were able to inhibit the proliferation of LNCaP cells, and the inhibitory effect of the combination was more significant than that of the individual drugs under the same conditions. The results showed that when 100 μmol/L SB and 200 μmol/L TRP were co-administered to LNCaP cells for 48 h, the inhibition rates of both were similar, and therefore the subsequent experiments were performed under this condition.

### 3.2 Silybin, triptorelin alone and in combination inhibit migration of LNCaP cells

To clarify the effects of SB and TRP alone and in combination inhibit migration of LNCaP cells, we used the concentrations of 100 μmol/L SB and 200 μmol/L TRP, alone and in combination, on LNCaP cells for 0, 24, 48, and 72h. The results showed that compared with the control group, the SB group, the TRP group, and the combination group all inhibited the migration of LNCaP cells; and the inhibitory effect of the combination group on the migration of LNCaP cells was more significant than that of the group using the drug alone (Fig 1d).

### 3.3 Silybin, triptorelin alone and in combination inhibit migration and invasion of LNCaP cells

We further investigated the effects of SB, TRP alone and in combination on LNCaP cell migration and invasion. Transwell results showed that LNCaP cell migration and invasion were inhibited in the SB group, TRP group and combination group compared with the control group, and the inhibition of LNCaP cell migration and invasion was more pronounced in the combination group (Fig 1e-1g).

### 3.4 Sample repetitive analysis

**Fig 2:**
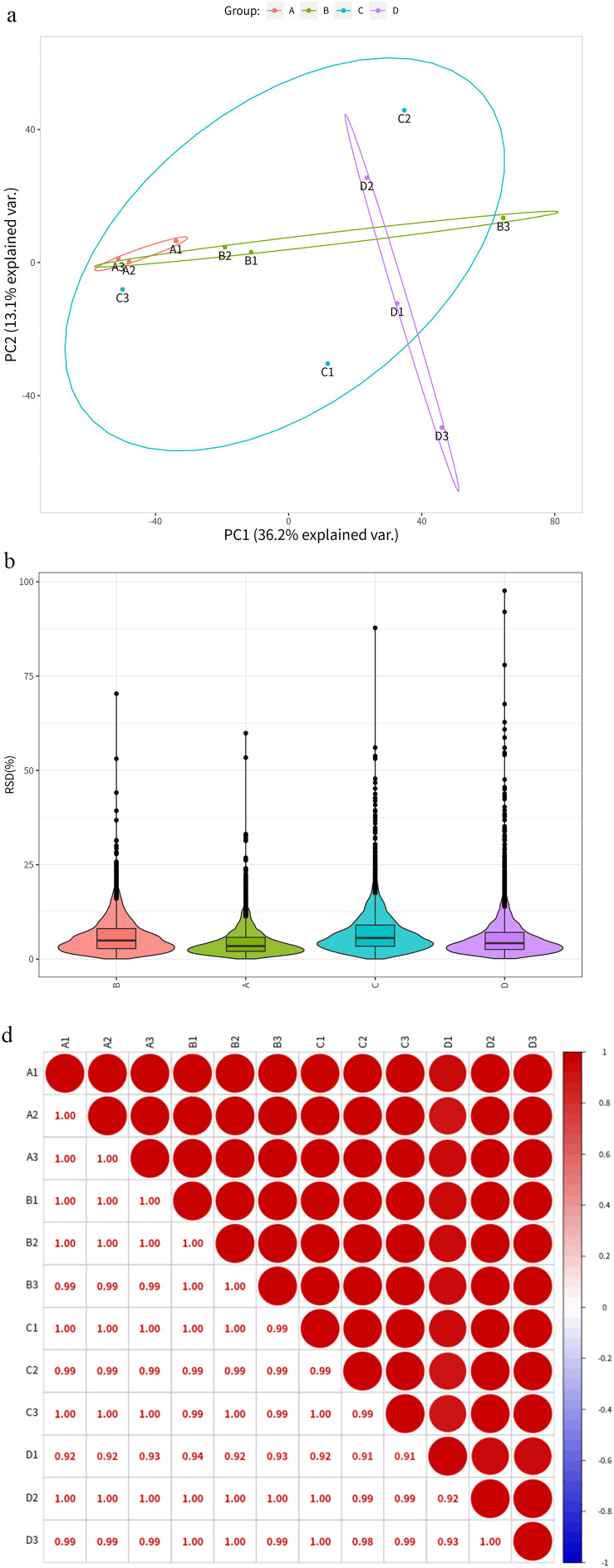
Systemic quality control of TMT-based quantitative proteomic data. **(a)** Principal components analysis of proteomic data across four groups. **(b)** Comparison of RSD of the four groups. **(c)** Matrix of Pearson correlation coefficients for the four groups. Groups A, B, C, and D represent the control, SB-treated, TRP-treated, and combination-treated groups, respectively.

In order to determine the protein expression changes caused by SB, TRP alone and in combination when acting on LNCaP cells, we first performed principal component analysis (PCA), as shown in Fig 2a, three samples from each group were clustered together, indicating good sample reproducibility. After that, we calculated the relative standard deviation (RSD) of each group, as shown in Fig 2b, the RSD was less than 20% in each group of samples, which also indicated reliable sample reproducibility. Finally, the Pearson correlation coefficients between the two samples of each group were calculated to draw a matrix, as shown in the Fig 2c, the redder the color, the stronger the positive correlation, and the bluer the color, the stronger the negative correlation, which again indicates that the four groups of protein samples have good reproducibility.

### 3.5 Quantitative proteomic analysis of differentially expressed proteins

**Fig 3:**
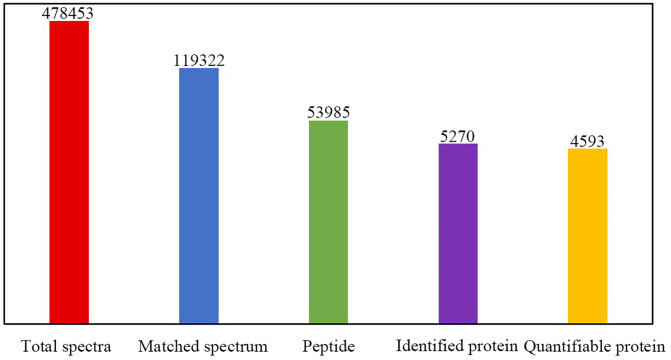
Statistical picture of identification and quantitative results.

In this experiment, a total of 478453 secondary profiles were identified by TMT quantitative proteomics analysis from LNCaP cells in control group, SB group, TRP group and combined group, of which the number of valid profiles was 119322, the number of peptides identified was 53985, and 5,270 proteins were identified, of which 4,593 proteins were quantified as shown in Fig 3.

### 3.6 Recognition of differentially expressed proteins

**Fig 4:**
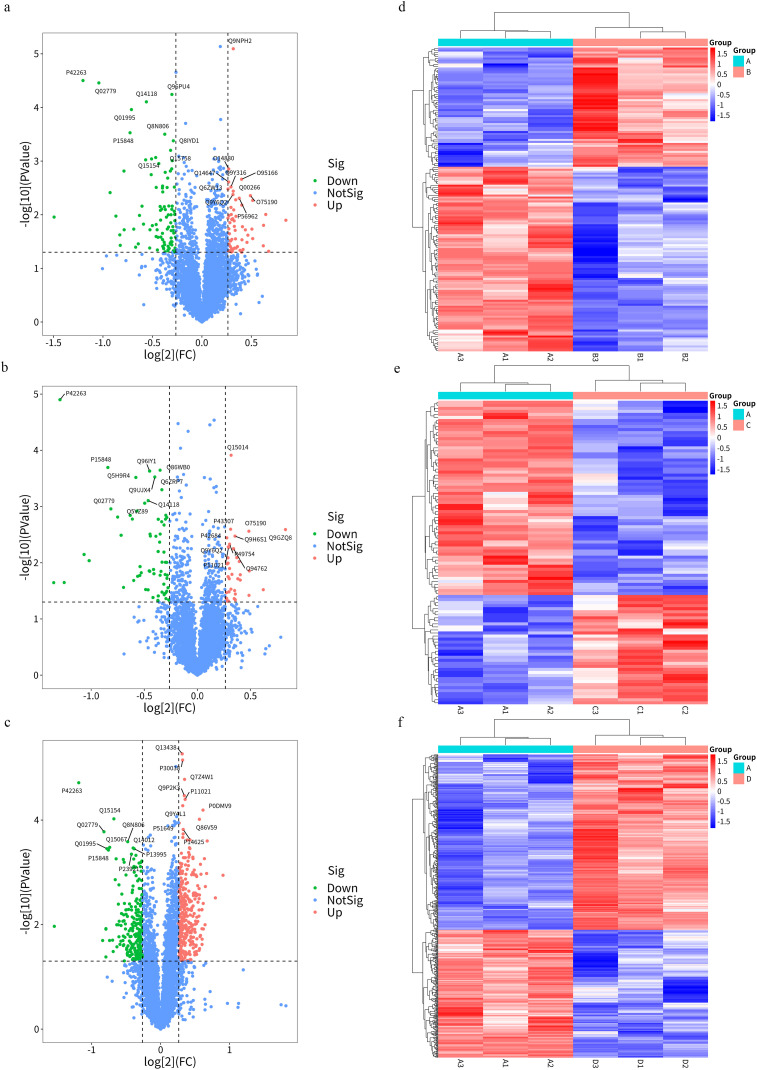
Volcano plots and heat maps showing the significantly DEPS. Volcano plots of DEPs identified from the A vs. B **(a)**, A vs. C **(b)** and A vs. D **(c)** comparisons. The red dots represent upregulated proteins, and the green dots represent downregulated proteins. Heat maps of DEPs identified from the A vs. B **(d)**, A vs. C **(e)** and A vs. D **(f)** comparisons. Groups A, B, C, and D represent the control, SB-treated, TRP-treated, and combination-treated groups, respectively.

The experimental data were further screened in order to analyze the DEPs between the different groups. DEPs were analyzed bioinformatically and showed that 153 DEPs (60 up-regulated and 93 down-regulated) were identified between the A vs. B comparison groups, 100 DEPs (36 up-regulated and 64 down-regulated) were identified between the A vs. C comparison groups,524 DEPs (303 up-regulated and 221 down-regulated) were identified between the A vs. D comparison groups, FC and *p*-values were screened for DEPs and represented by volcano plots (Fig 4a-4c). Cluster analysis revealed significant differences in the data patterns between the comparison groups and a high degree of similarity among the three biological replication groups within each group, indicating that there were significant differences in protein expression levels between each of the two comparison groups, and that the DEPs were able to represent samples that were significantly affected by the biological treatments (Fig 4d-4f). Table 1 summarizes all DEPs in each group.

**Table 1:** Number of DEPs in each group.

Compared groups	Up-regulated	Down-regulated	Total difference
A vs. B	60	93	153
A vs. C	36	64	100
A vs. D	303	221	524

### 3.7 Bioinformatics analysis of differentially expressed proteins in each group

#### 3.7.1 GO.

**Fig 5:**
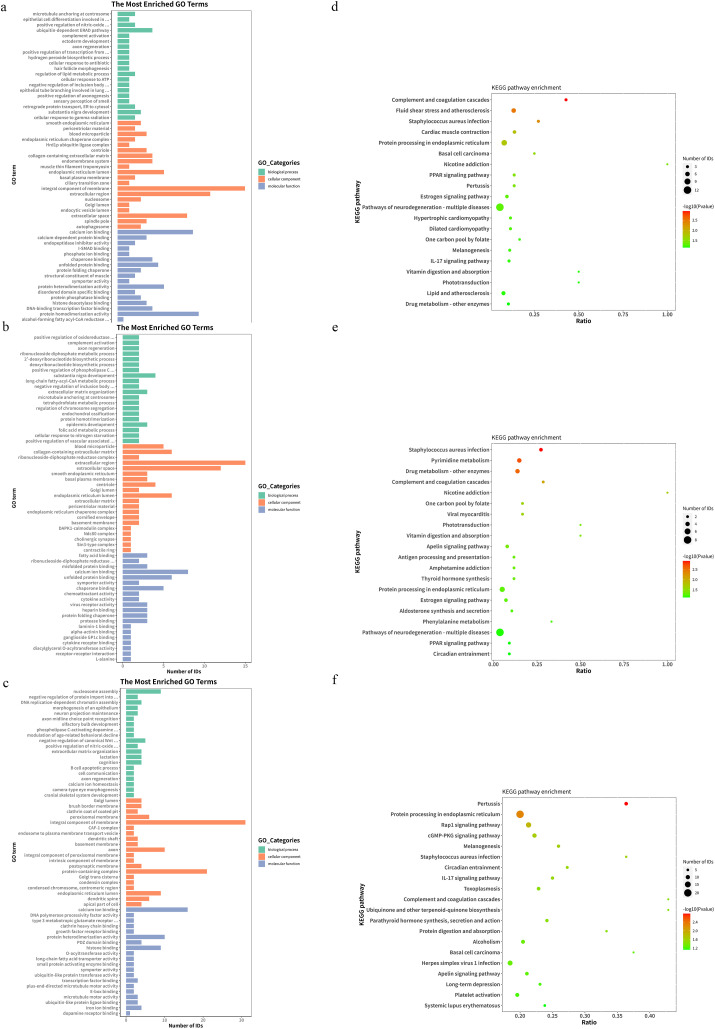
GO functional classification and KEGG pathway enrichment analysis of DEPs. The 3 bar charts show the GO functional classification of the DEPs identified from the A vs. B **(a)**, A vs. C **(b)** and A vs. D **(c)** comparisons. Enrichment of KEGG pathways with DEPs identified from the A vs. B **(d)**, A vs. C **(e)** and A vs. D **(f)** comparisons. Groups A, B, C, and D represent the control, SB-treated, TRP-treated, and combination-treated groups, respectively.

Based on the Gene Ontology (GO) database, we investigated the enrichment of DEPs in LNCaP cells under three different culture situations. The DEPs were associated with 1696, 1111, and 3805 functional annotations in the A vs. B, A vs. C, and A vs. D comparison groups, respectively. The vocabulary of genes and gene products involved in GO is categorized into three main groups covering three aspects of biology: biological process (BP), cellular component (CC), and molecular function (MF). Fig 5a-5c shows the top 20 significantly enriched terms in each otology: biological processes, cell components, and molecular functions.

#### 3.7.2 KEGG.

To further understand the signaling pathways in which the differential proteins between the comparison groups may be involved, we performed pathway enrichment analysis of these differential proteins using KEGG. Analysis shows that the A vs. B comparison group achieved enrichment in pathways such as IL-17 signaling pathway, Pathways of neurodegeneration – multiple diseases, Protein processing in endoplasmic reticulum, Fluid shear stress and atherosclerosis and Estrogen signaling pathway; the A vs. C comparison group achieved enrichment in pathways such as Apelin signaling pathway, Circadian entrainment, Pathways of neurodegeneration – multiple diseases, Protein processing in endoplasmic reticulum and Pyrimidine metabolism; the A vs. D comparison group achieved enrichment in pathways such as IL-17 signaling pathway, Circadian entrainment, Apelin signaling pathway, Protein processing in endoplasmic reticulum and Rap1 signaling pathway. Fig 5d-5f shows the top 20 KEGG pathways in the list of significance.

Notably, in the A vs. B and A vs. D comparison groups, more DEPs were enriched in the same signaling pathway (IL-17 signaling pathway) (Table 2). Of note, in the A vs. C and A vs. D comparison groups, some of the same DEPs were enriched for two identical signaling pathways (Circadian entrainment and Apelin signaling pathway) (Table 3). These results suggest that the SB and TRP combination group may regulate the bone metabolic profile of LNCaP cells through these signaling pathways and DEPs.

**Table 2:** DEPs from the A vs. B and A vs. C comparisons in the IL-17 signaling pathway.

Compared groups	DEPs of the IL-17 signaling pathway
A vs. B	RELA, HSP90B1, ANAPC5
A vs. D	RELA, HSP90B1, MAPK1, MAP3K7, MAPK4, MAPK15, CASP3

**Table 3:** DEPs from the A vs. C and A vs. D comparisons.

Compared groups	DEPs of the Circadian entrainment	DEPs of the Apelin signaling pathway
A vs. C	GRIA3, CALM3	MEF2D, MAP1LC3B
A vs. D	GRIA3, CALM3, MAPK1, GNAI1, GNAI3	MEF2D, MAP1LC3B, MAPK1, GNAI1, GNAI3

#### 3.7.3 PPI.

**Fig 6:**
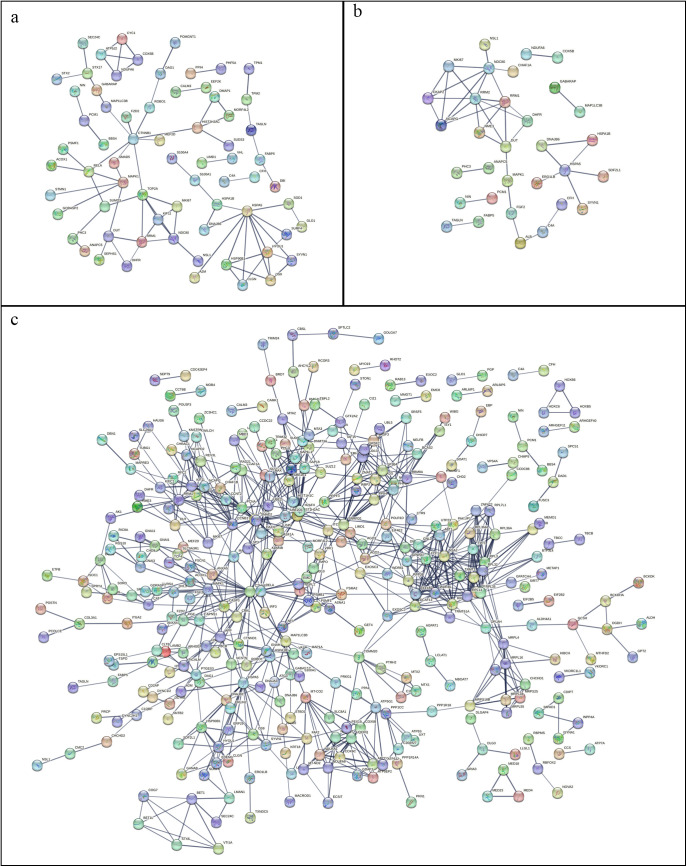
Protein-interaction network of DEPs in the A vs. B. **(a), A vs. C (b) and A vs. D (c) comparisons.** Groups A, B, C, and D represent the control, SB-treated, TRP-treated, and combination-treated groups, respectively.

Protein network interactions map can visualize the correlation between proteins and proteins, based on PPI analysis Fig 6a-6c, we found that the number of DEPs in the combination group was significantly increased, and the interactions between the proteins were more obvious and more closely linked.

### 3.8 Validation of differentially expressed proteins by Western blot

**Fig 7:**
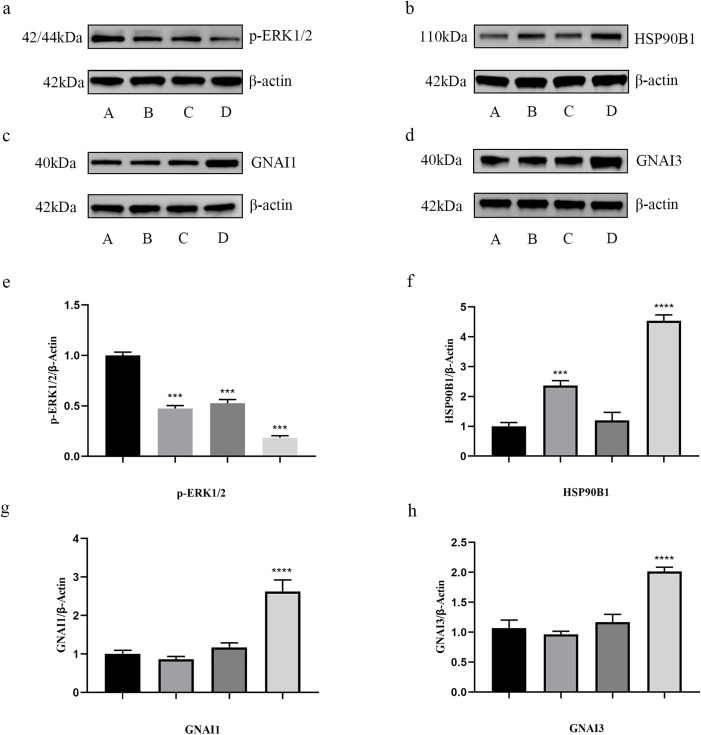
Validation of DEPs identified from the comparisons of the A vs. B, A vs. C and A vs. D comparisons. Western blot analysis of proteins including p-ERK2 **(a, e)**, HSP90B1 **(b, f)**, GNAI1 (c, g) and GNAI3 **(d, h)**. The data are shown as the mean ± SD values; ***P < 0.001, ****P < 0.0001, vs. untreated control group. Groups A, B, C, and D represent the control, SB-treated, TRP-treated, and combination-treated groups, respectively.

To validate the accuracy of the proteomics results, we selected four DEPs (p-ERK2, HSP90B1, GNAI1, and GNAI3) and detected their expression levels by Western blot. As shown in Fig 7, compared with group A, p-ERK2 expression decreased in groups B, C, and D, with the most pronounced reduction observed in group D (*P* < 0.001). HSP90B1 expression increased in both groups B and D, with a more pronounced increase in the combination therapy group (*P* < 0.0001). In group C, HSP90B1 expression showed no significant change (*P* > 0.05). The expression levels of GNAI1 and GNAI3 showed no significant changes in groups B and C (*P* > 0.05). However, the expression levels of GNAI1 and GNAI3 significantly increased in group D (*P* < 0.0001). The Western blot results were consistent with the proteomics findings.

## 4. Discussion

To the best of our knowledge, this is the first time that this approach has been used to explore the potential molecular mechanisms underlying the effects of SB combined with TRP on bone metabolism in LNCaP cells. In this study, with the help of TMT-based proteomics analysis, we identified 153 DEPs, 100 DEPs, and 524 DEPs from the three comparison groups of A vs. B, A vs. C, and A vs. D, respectively. These DEPs play crucial roles in the combined action of SB and TRP on LNCaP cells and may be closely related to LNCaP cell bone metabolism is closely related.

In order to better explore the role of these DEPs, we performed further bioinformatics analysis, and found that a higher number of DEPs were enriched in the IL-17 signaling pathway in both A vs. B and A vs. D comparative groups. The IL-17 signaling pathway is a typical inflammatory pathway, and the IL-17 family is composed of IL- 17A, IL-17B, IL17C, IL-17D, IL-17E, and IL-17F, which play key roles in acute and chronic inflammatory responses [45]. IL-17 is produced by T helper cells (Th17 cells) and is a potent regulator of bone homeostasis [46]. IL-17A is the prototypical IL-17 family and IL-17F has the highest homology to IL -17A has the highest homology, and in recent studies it has been shown that their activated expression is closely related to bone metabolism. TRAF3 (TNF receptor-associated factor 3) as an inhibitor of osteoclastogenesis, enhances osteoclastogenesis when it is degraded [47,48], and during the receptor activator of NF-κB ligand (RANKL)-induced osteoclastogenesis, high levels of IL-17A prevent the degradation of TRAF3, which in turn promotes osteoclast apoptosis [49], while osteoclastogenesis can also be blocked at high levels of IL-17A by activating caspase 3 to promote its overexpression [50]. It has also been found that when osteoblasts from neonatal rats were treated with different concentrations of IL-17F, IL-17F was able to significantly promote osteoblast proliferation, differentiation and mineralization [51]. Together with our findings, these results support that the combination medication may inhibit the abnormal bone metabolism of LNCaP cells through the IL-17 signaling pathway.

RELA (P65) is one of the members of the NF-κB/REL family, which consists of five members: p50, p52, p65, c-REL and REL-B proteins, which form homodimers or heterodimers and are important regulators of immune and inflammatory responses [52]. Studies have shown that RELA is expressed in mouse growth plates and it is able to promote longitudinal bone growth and growth plate cartilage formation by inducing bone morphogenetic protein-2 (BMP-2) expression, in addition to mediating the growth-promoting effects of insulin-like growth factor-1 (IGF-1) on chondrogenesis and longitudinal bone growth [53]. By inhibiting the activation of RELA, it can block RANKL-induced osteoclast development and bone resorption process, which can have a positive effect on the treatment of bone diseases with excessive bone resorption [54]. RELA can also bind to the promoter region of the C-X-C chemokine receptor type 4 (CXCR4) gene to activate its transcription, which will lead to an increase in the expression of CXCR4 protein, and CXCR4 induces an increase in osteoblast proliferation [55]. Therefore, we hypothesized that SB might regulate TRP-induced bone metabolism abnormalities by elevating the expression level of RELA to regulate bone metabolism-related factors.

HSP90b1 is a byproduct of heat shock protein 90 (HSP90) in the endoplasmic reticulum, often also known as grp94 [56]. Heat shock proteins (HSPs) are a class of functionally related molecular chaperones involved in a variety of biological processes such as protein folding, assembly and transport, peptide transport and antigen presentation. Grp94 is associated with Wnt signaling [57]. Low-Density Lipoprotein Receptor-Related Protein 6 (LRP6) is a co-receptor for the cell surface Wnt receptor Frizzled, which is required for typical Wnt signaling [58], LRP6 expression at the cell surface is dependent on grp94, and when grp94 is deficient, LRP6 cannot be exported from the endoplasmic reticulum to the cell surface, resulting in a severe loss of typical Wnt signaling [59]. And when Wnt/β-catenin signaling is inhibited it leads to reduced osteogenic activity [60]. Grp94 is also a marker protein for endoplasmic reticulum stress and is essential for stabilizing the endoplasmic reticulum internal environment during cellular stress [61]. Endoplasmic reticulum stress can lead to overexpression of the intrinsic endoplasmic reticulum calcium-binding protein calreticulin, which has been shown to regulate and reduce calcium flux in the endoplasmic reticulum [62–64], and the reduction of osteoclast differentiation caused by calcium flux is closely related to the expression of calreticulin. It has been found [65], that endoplasmic reticulum stress also induces the expression of cysteine-rich with EGF-like domains2 (CRELD2) gene, which plays an important role in bone development and endosteal homeostasis by promoting the differentiation of chondrocytes and osteoblasts [66]. CRELD2 expression blocks calcium release from the endoplasmic reticulum, resulting in a decrease in calcium-dependent calmodulin neurophosphatase activity, which in turn affects the nuclear translocation of Nuclear Factor of Activated T-cells, Cytoplasmic 1(NFATc1), ultimately leading to impaired osteoclast differentiation [67]. Our study found that the high expression of grp94 in the SB and TRP combination group may have a beneficial effect on the regulation of bone metabolism.

Mitogen-activated protein kinase 1 (MAPK1) is also known as extracellular signal regulated protein kinase 2 (ERK2). In the MARK family, ERK is the main link in its signaling and includes two isoforms, ERK1 and ERK2. MAPK1 is the core of the Ras-MAKK signaling pathway, and when MAPK1 is activated, it can conduct signals into the nucleus, activate a variety of transcription factors by phosphorylating them in the nucleus, and promote and regulate the expression of some genes related to cell growth and differentiation [68]. In vertebrates, chondrocytes undergo a series of proliferation and differentiation eventually ossifying to form long bones [69]. During this process, when early hypertrophic chondrocytes are transformed into terminally differentiated chondrocytes, its positive regulators are ERK1 and ERK2, and Egr1 and Egr2 mediate this transformation process, and when ERK1 and ERK2 are absent in hypertrophic chondrocytes, the expression of Egr1 and Egr2 is down-regulated, which leads to impaired terminal differentiation of chondrocytes, and ultimately leads to delayed endochondral ossification [70]. ERK1 and ERK2 are also important in the process of osteoblast differentiation, and ERK1 and ERK2 increase MAPK signaling in mesenchymal cells, leading to enhanced osteoblast differentiation [71]. Therefore, we suggest that the expression of MAPK1 in the combined group may be beneficial in regulating abnormal bone metabolism.

We elaborated on three of these DEPs, of which RELA and HSP90b1 were the same DEPs in the A vs. B and A vs. D comparator groups, whereas MAPK1 was a new DEP added to the combination group. We hypothesize that in the combination group, TRP will act on LNCaP cells and exert a negative regulatory effect on bone metabolism. Concurrently, SB will activate the positive regulation of bone metabolism. As a result, a greater number of proteins beneficial to the regulation of bone metabolism are likely to be expressed in the combination group.

To better analyze the effects of the combination group on bone metabolism in LNCaP cells, we also compared two comparison groups, A vs. C and A vs. D, whose DEPs were enriched to two identical signaling pathways, Circadian entrainment and Apelin signaling pathway. More DEPs were enriched in the Circadian entrainment and Apelin signaling pathway in comparison group A vs. D compared to comparison group A vs. C. Surprisingly, the DEPs enriched in both signaling pathways were GNAI1, GNAI3.

The G alpha inhibitory subunit (Gαi) was originally named for its ability to inhibit adenylate cyclase activity, and Gαi1 and Gαi3 are encoded by the GNAI1 and GNAI3 genes [72]. Gαi is a member of the G protein family, which is involved in the pathology of a wide range of diseases. G proteins play a crucial role in bone development and reconstruction, as well as in various bone and joint diseases, and abnormalities in G protein structure can impair bone formation and bone homeostasis [73]. Activation of Gαi results in the inhibition of adenylate cyclase (AC), which leads to a decrease in the production of cyclic adenosine monophosphate (cAMP). It has been found that Gαi1 and Gαi3 are involved in the biological functions of several growth factors, linking cell surface receptors to intracellular effectors, which in turn are involved in vascular regeneration and repair of nerve damage. Lutz Birnbaumer et al [72] found that deletion or aberrant expression of Gαi1 and Gαi3 resulted in abnormal skeletal development in mice, with particularly pronounced effects on lumbar spine and rib development. This suggests that Gαi1 and Gαi3 play important roles in bone development and stabilization. In the present study, we found that GNAI1 and GNAI3 expression was up-regulated by the combined group in LNCaP cells, and we hypothesized that the combined effect might regulate TRP-induced bone metabolism abnormality in LNCaP cells by regulating GNAI1 and GNAI3.

## 5. Conclusions

In short, our findings suggested that SB could regulate TRP-induced bone metabolism abnormalities in LNCaP cells. The proteomics and bioinformatics analysis revealed that SB regulates bone metabolism abnormalities mainly through IL-17 signaling pathway. Five key proteins, p-ERK2, RELA, HSP90B1, GNAI1, and GNAI3, are also important regulators to ameliorate this abnormality and their specific functions need to be further studied. Our results offered novel information about the underlying molecular mechanisms involved in SB to regulate TRP-induced bone metabolism abnormality in LNCaP cells, and also provide a new direction and theoretical basis for clinical treatment of PCa.

## Supporting information

S1 File Minimal data set. (XLSX)

S2 File Western blot 3. (DOCX)
