# Supplementary figures and images for: Effects of silybin on triptorelin-induced bone metabolic abnormalities in prostate cancer revealed based on TMT-based proteomics
Source: PLoS One. 2026 Jan 29;21(1):e0341064. doi: 10.1371/journal.pone.0341064 (PMC12854432; doi:10.1371/journal.pone.0341064)

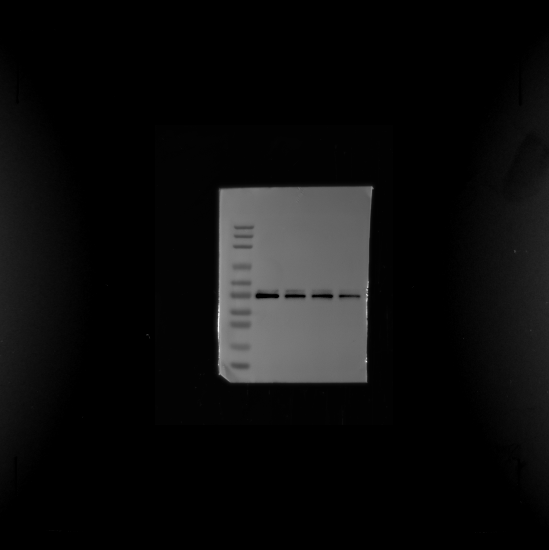

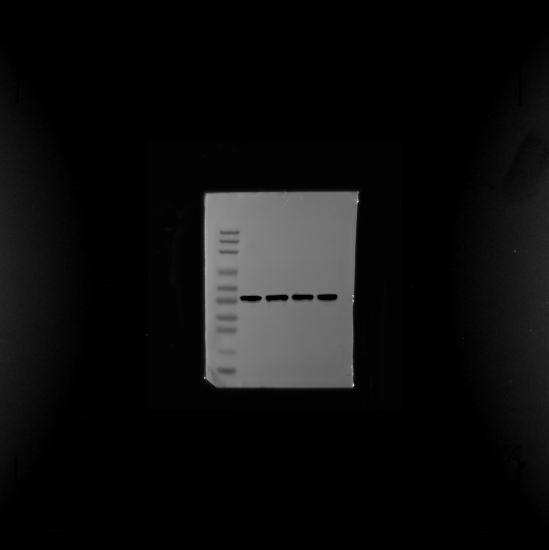


ACTIN p-ERK


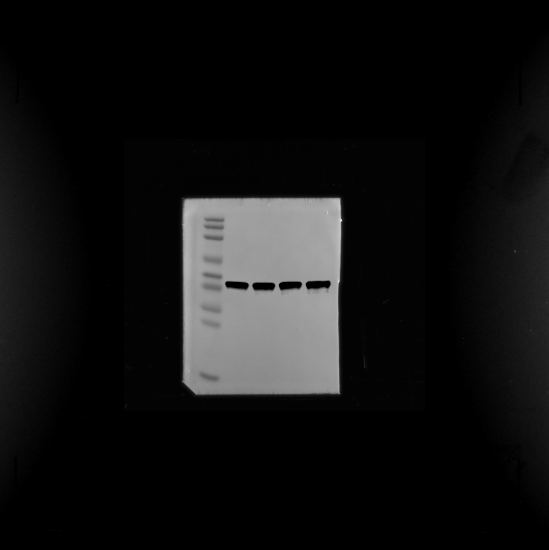

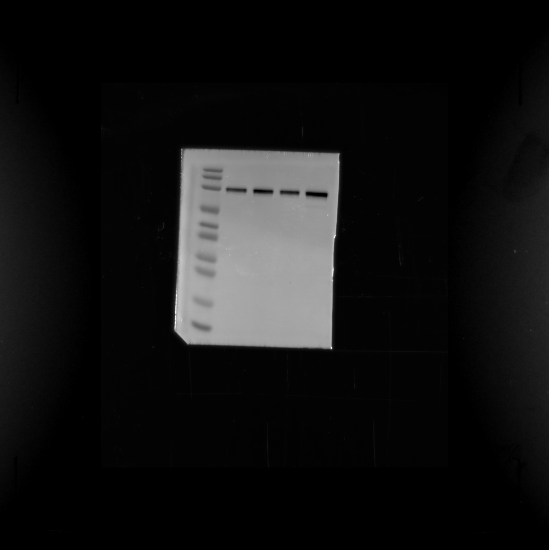


ACTIN HSP90B1


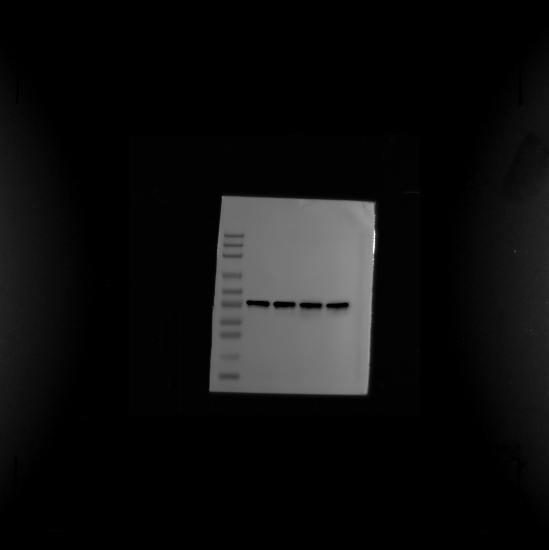

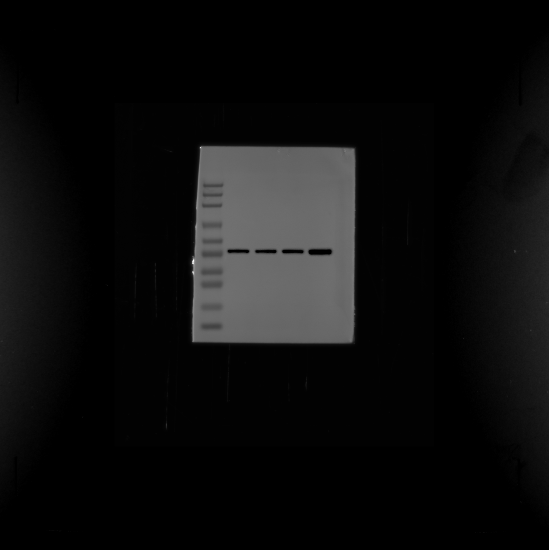


ACTIN GNAI1


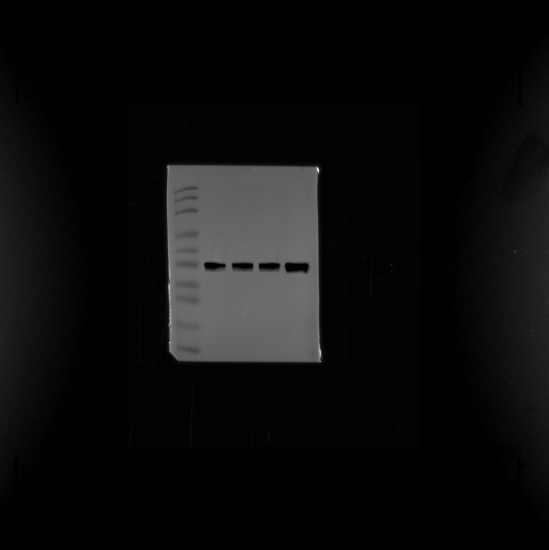

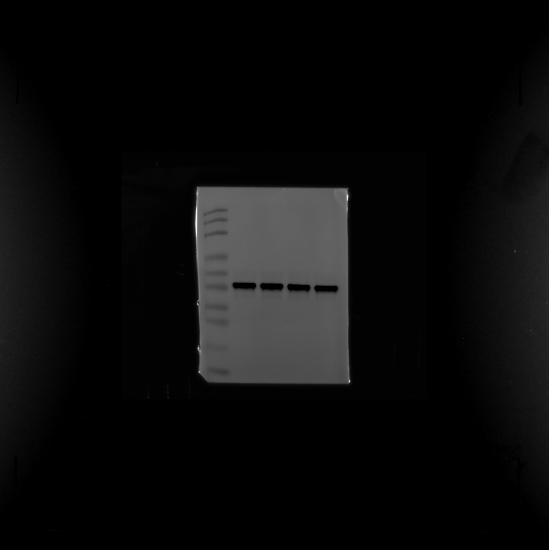


ACTIN GNAI3


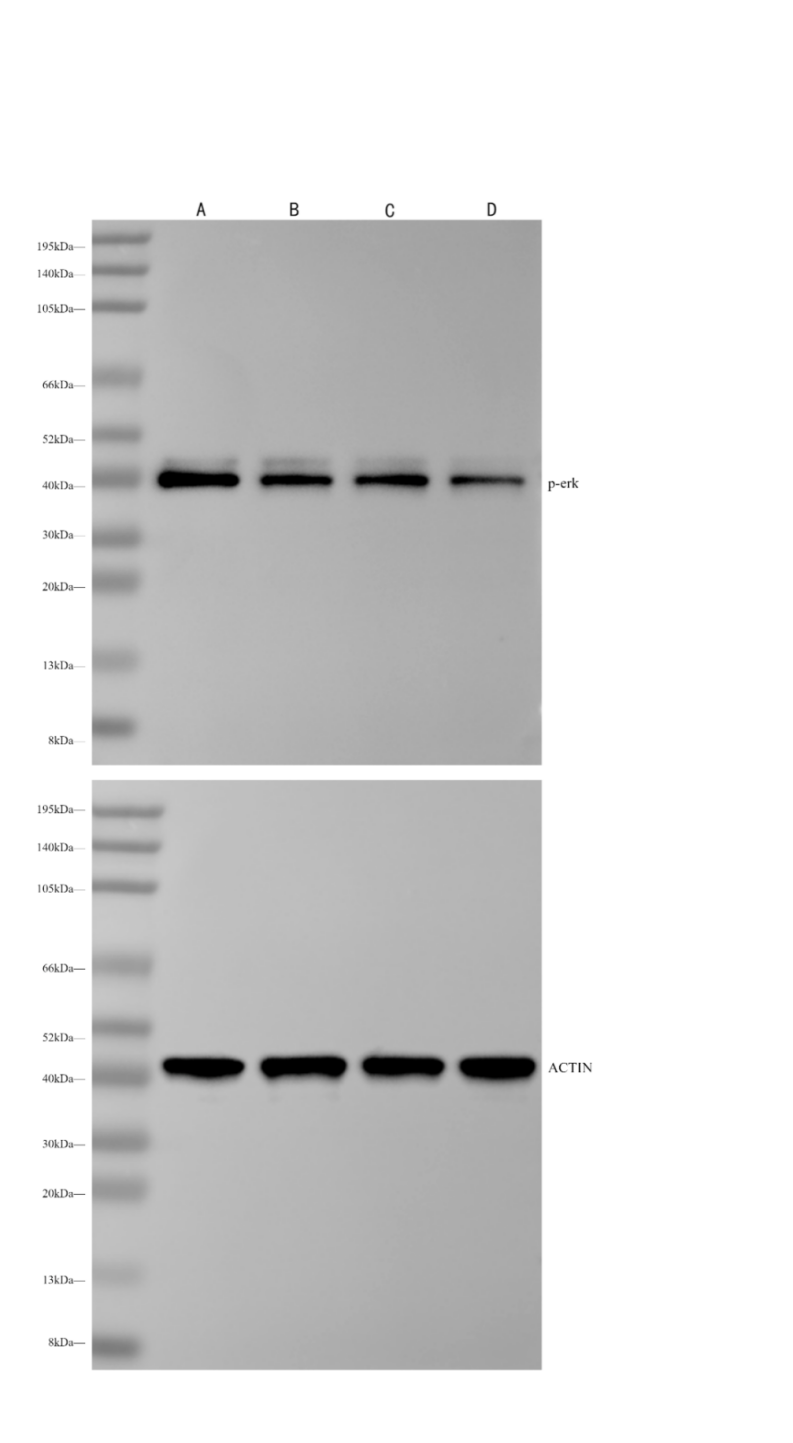

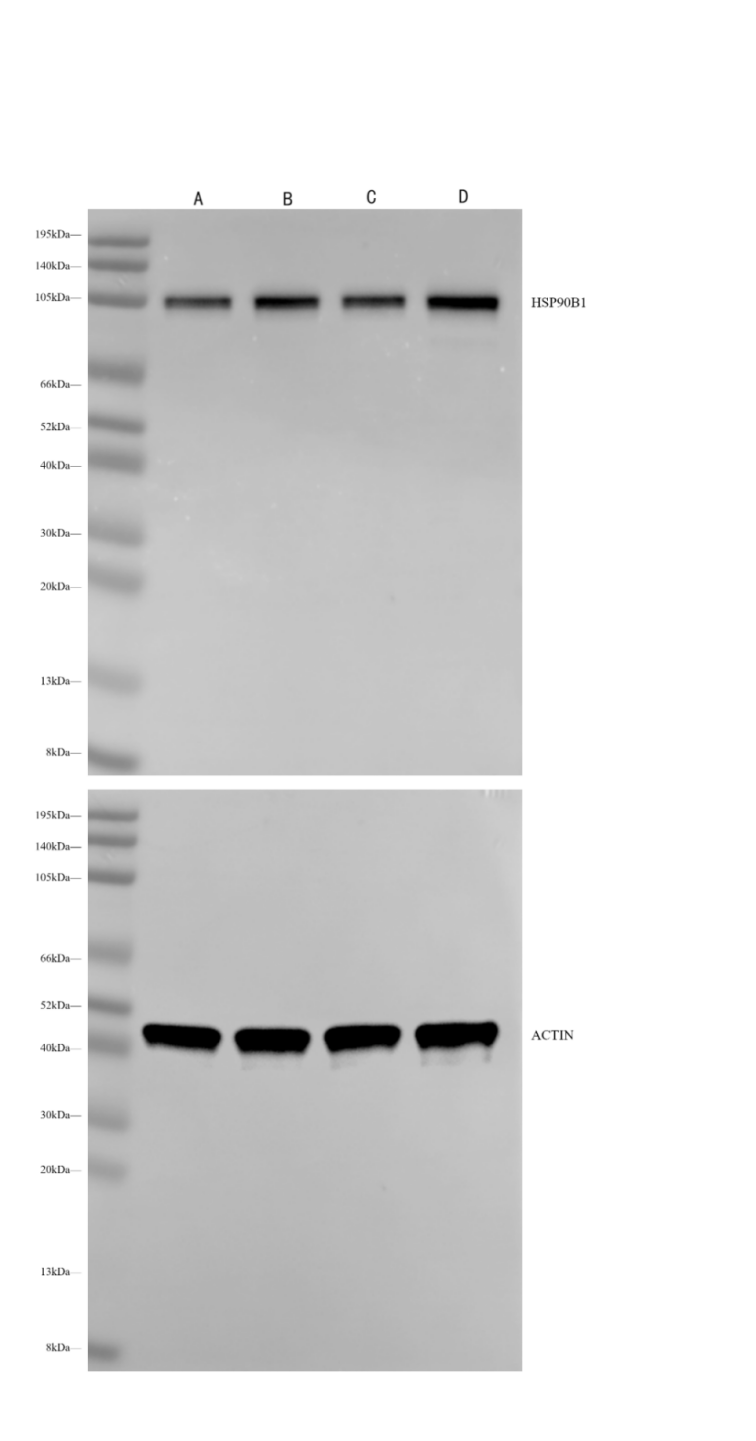


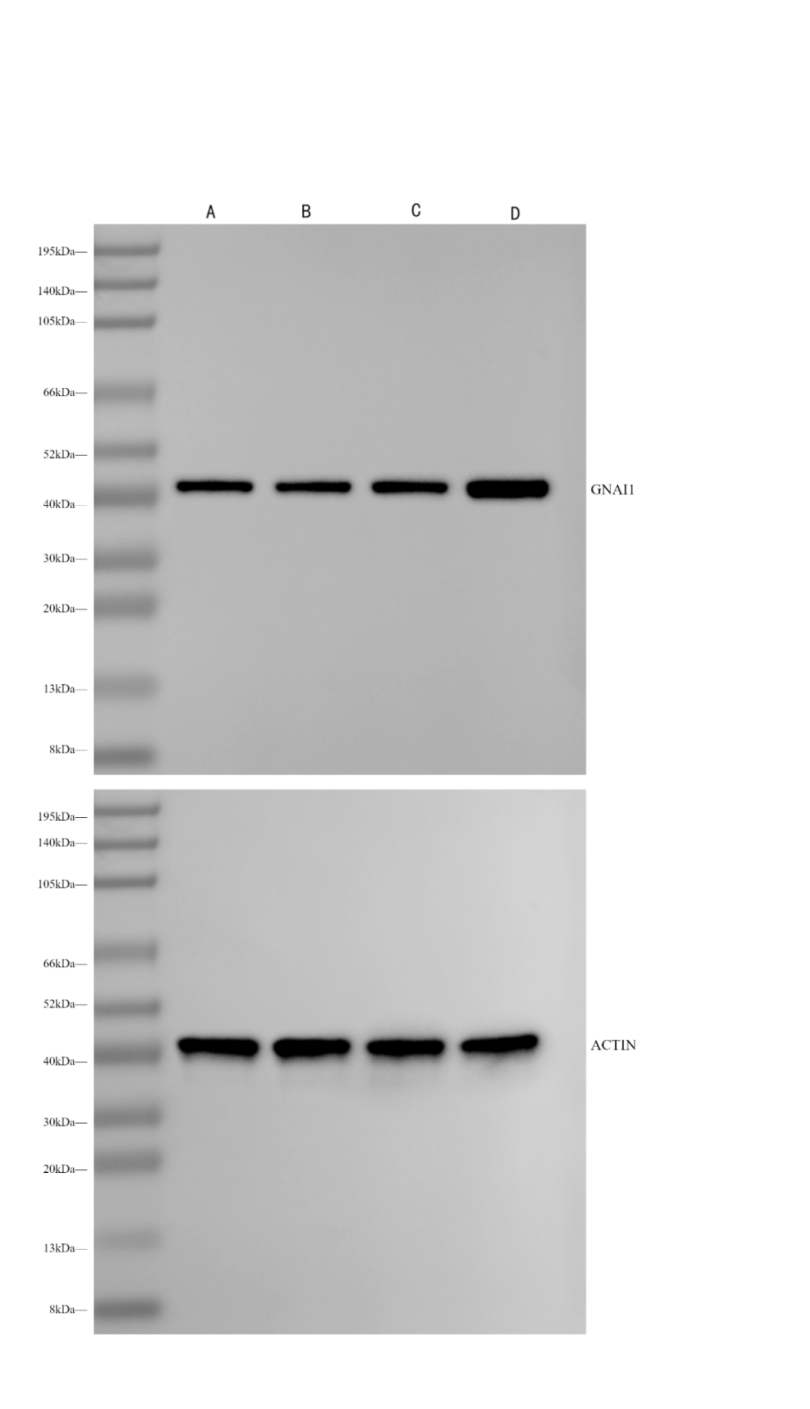

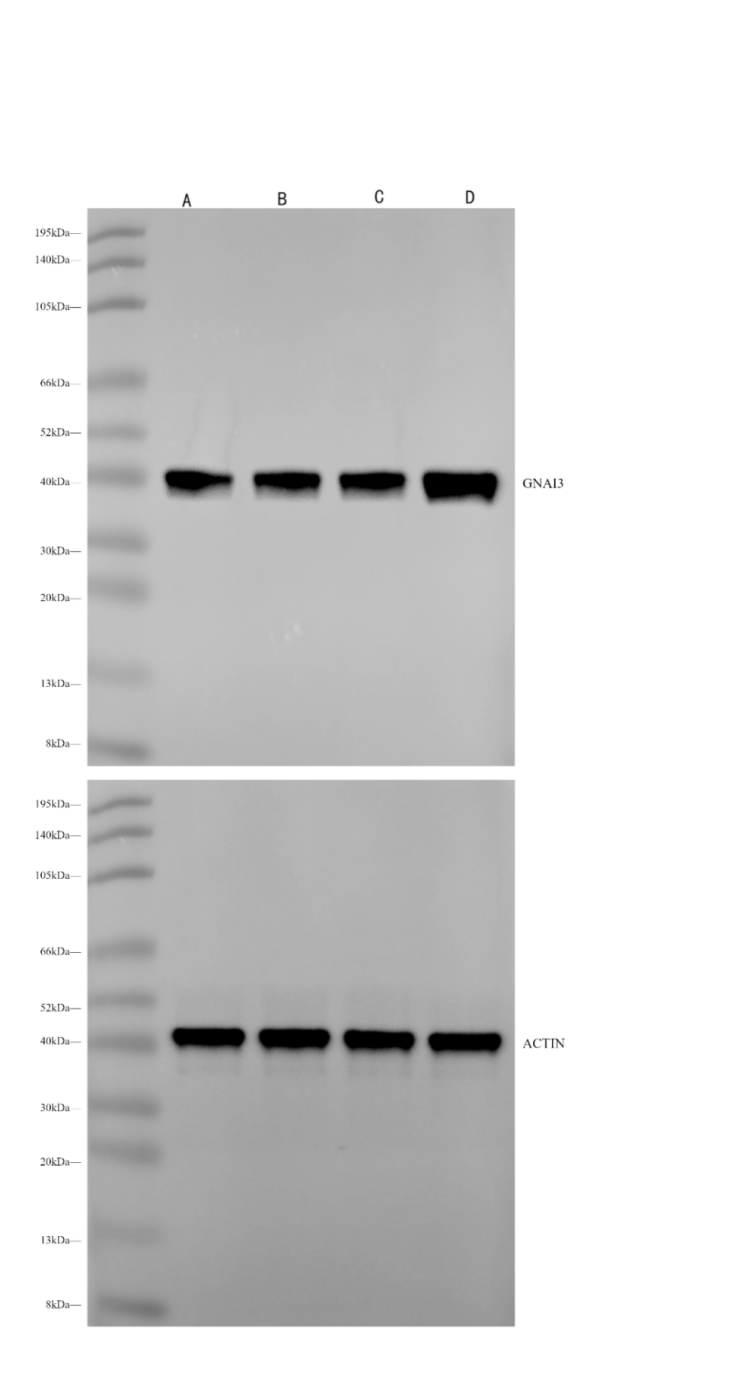

Supplement: S2 File — (DOCX) [file pone.0341064.s002.docx]
